# Mechanisms of regulation of SNF1/AMPK/SnRK1 protein kinases

**DOI:** 10.3389/fpls.2014.00190

**Published:** 2014-05-20

**Authors:** Pierre Crozet, Leonor Margalha, Ana Confraria, Américo Rodrigues, Cláudia Martinho, Mattia Adamo, Carlos A. Elias, Elena Baena-González

**Affiliations:** ^1^Instituto Gulbenkian de CiênciaOeiras, Portugal; ^2^Escola Superior de Turismo e Tecnologia do Mar de Peniche, Instituto Politécnico de LeiriaPeniche, Portugal

**Keywords:** SnRK1, SNF1, AMPK, stress, energy signaling, *Arabidopsis*, kinase regulation

## Abstract

The SNF1 (sucrose non-fermenting 1)-related protein kinases 1 (SnRKs1) are the plant orthologs of the budding yeast SNF1 and mammalian AMPK (AMP-activated protein kinase). These evolutionarily conserved kinases are metabolic sensors that undergo activation in response to declining energy levels. Upon activation, SNF1/AMPK/SnRK1 kinases trigger a vast transcriptional and metabolic reprograming that restores energy homeostasis and promotes tolerance to adverse conditions, partly through an induction of catabolic processes and a general repression of anabolism. These kinases typically function as a heterotrimeric complex composed of two regulatory subunits, β and γ, and an α-catalytic subunit, which requires phosphorylation of a conserved activation loop residue for activity. Additionally, SNF1/AMPK/SnRK1 kinases are controlled by multiple mechanisms that have an impact on kinase activity, stability, and/or subcellular localization. Here we will review current knowledge on the regulation of SNF1/AMPK/SnRK1 by upstream components, post-translational modifications, various metabolites, hormones, and others, in an attempt to highlight both the commonalities of these essential eukaryotic kinases and the divergences that have evolved to cope with the particularities of each one of these systems.

## INTRODUCTION

The yeast SNF1 (sucrose non-fermenting 1), mammalian AMPK (AMP-activated protein kinase), and plant SnRK1 (SNF1-related protein kinase 1) are metabolic sensors belonging to a highly conserved eukaryotic protein kinase family (Hardie, 2007; Polge and Thomas, 2007; Hedbacker and Carlson, 2008). In yeast, SNF1 plays a fundamental role in the shift from fermentative to oxidative metabolism in response to glucose deprivation, partly by releasing the repression of genes essential for the utilization of alternative carbon sources (Hedbacker and Carlson, 2008). AMPK, the mammalian counterpart, is activated by a raise in the “adenylate charge,” i.e., a raise in AMP and ADP levels relative to ATP upon glucose starvation caused by fasting, exercise, or stresses like heat shock and hypoxia. Once activated, AMPK implements an energy-saving program through direct enzyme regulation and transcriptional control (Hardie, 2007). Anabolic processes such as the synthesis of fatty acids, cholesterol, and proteins are switched off, while catabolic pathways such as fatty acid oxidation, glycolysis, and autophagy are activated. AMPK plays also a broader role in regulating whole-body energy metabolism and glucose homeostasis through the regulation of processes like muscle glucose uptake, insulin production and secretion, management of body lipids, and appetite (Hardie et al., 2012b).

The first piece of evidence on the functional conservation of the plant ortholog and the regulation of energy metabolism by SnRK1 came from the complementation of the yeast *snf1*Δ mutant with a rye Snf1-related cDNA. Complementation reestablished the utilization of non-fermentable carbon sources such as ethanol and glycerol, indicating that rye Snf1-related cDNA could substitute Snf1 in the sugar signaling pathway (Alderson et al., 1991). Similar results were obtained in yeast complementation assays using SnRK1 from other plant species, such as tobacco, potato, and *Arabidopsis* (Muranaka et al., 1994; Bhalerao et al., 1999; Lovas et al., 2003b).

The large *Arabidopsis* SnRK super family is composed of three distinct subfamilies, SnRK1, SnRK2, and SnRK3 (Hrabak et al., 2003). The SnRK2 and SnRK3 subfamilies include 35 more divergent protein kinases specific to plants and mostly known for their involvement in stress and abscisic acid (ABA) signaling (Weinl and Kudla, 2009; Umezawa et al., 2010). The SnRK1 subfamily comprises SnRK1α1/SnRK1α2/SnRK1α3 (also named SnRK1.1/SnRK1.2/SnRK1.3, AKINα1/AKINα2/AKINα3, KIN10/KIN11/KIN12, or AKIN10/AKIN11/AKIN12), the catalytic subunits of the SnRK1 complex and the closest relatives of Snf1 and AMPKα. Of these, only SnRK1α1/SnRK1α2 appear to be expressed (Baena-Gonzalez et al., 2007). The gene family in cereals comprises two subgroups, of which SnRK1a is more closely related to the homolog present in dicots and SnRK1b is cereal-specific and mostly expressed in the seed (Halford and Hardie, 1998).

SnRK1 regulates metabolism and transcription in response to energy deprivation and ABA signals, and is inactivated by sugars that restore an energy balance (Polge and Thomas, 2007; Baena-Gonzalez and Sheen, 2008; Rodrigues et al., 2013). Mounting evidence indicates that SnRK1 plays a crucial role in the acclimation of plants to a wide range of biotic and abiotic stresses (Hao et al., 2003; Lovas et al., 2003a; Schwachtje et al., 2006; Baena-Gonzalez et al., 2007; Lee et al., 2009).

Besides their role in metabolism and stress responses these kinases regulate virtually all aspects of cell function as well as multiple developmental processes. Consistent with the established role of SNF1 and AMPK in the control of cell growth and proliferation, SnRK1 was recently shown to regulate cell cycle progression (Guerinier et al., 2013). In addition, AMPK regulates cell polarity and apoptosis and SNF1 regulates yeast-specific processes like mating and sporulation (Hedbacker and Carlson, 2008; Hardie, 2011; Carling et al., 2012). In plants, SnRK1 was shown to be crucial for seed filling and maturation, and to affect embryo development and cotyledon growth (Radchuk et al., 2006, 2010), as well as pollen development (Zhang et al., 2001), lateral organ development and phase transition (Tsai and Gazzarrini, 2012).

**FIGURE 1:**
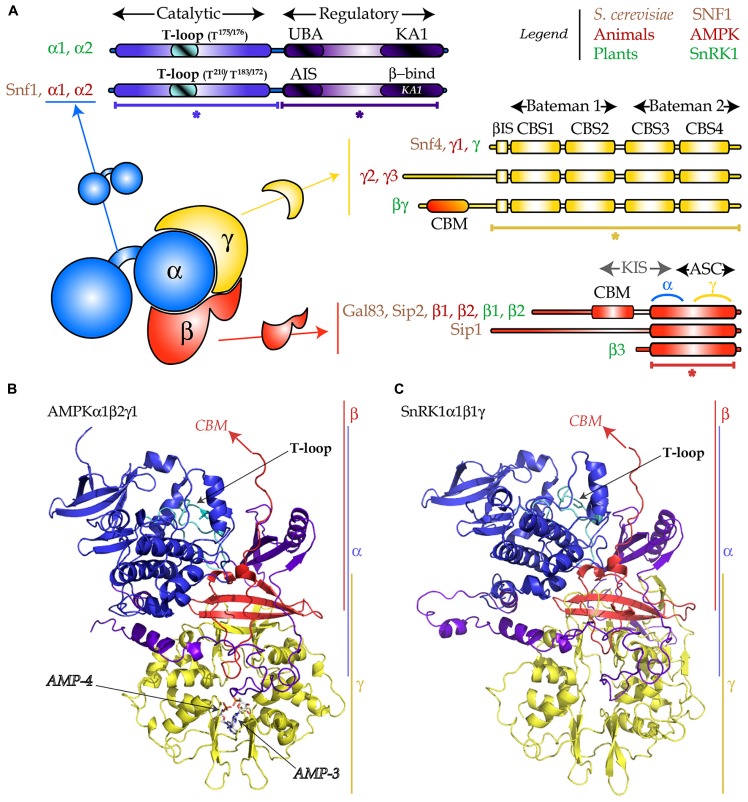
**Heterotrimeric structure of the SNF1/AMPK/SnRK1 complexes. (A)** The α-subunit (in blue) is composed of a catalytic domain (in blue with the T-loop in cyan) and a regulatory domain (in purple-blue) which encompasses an auto-inhibitory sequence (AIS) or an ubiquitin-associated (UBA) domain, and a kinase-associated (KA1) domain for binding the β- and γ-subunits. The γ-subunit (in yellow) is composed of two “Bateman” domains each of them containing two CBS (cystathionine-β-synthase) domains and a β-interacting sequence (βIS). The AMPKγ2 and γ3 bear an N-terminal extension and the plant-specific SnRK1βγ possesses a carbohydrate binding module (CBM). The β-subunit (in red) harbors an ASC (association to the complex) domain, containing the sites of interaction with γ and α, a CBM and an N-terminal extension. The KIS (kinase interacting sequence) domain, traditionally used for designating the region comprising the CBM and the site for interaction with the α-subunit, is no longer used. The plant-specific SnRK1β3 is atypical as it does not possess the CBM or the N-terminal extension. **(B)** Cartoon representation of the 3D-structure (PDB: 2Y94) of the AMPKα1β2γ1 complex. Asterisks designate parts in **(A)** that were crystallized. Arrows indicate missing parts (CBM), the T-loop, and the two AMP molecules. **(C)** 3D-structure model of SnRK1α1β1γ, generated with Swiss-Model using as template the AMPK structure presented in **(B)**. Color code in **(B,C)** as described in **(A)**.

**FIGURE 2:**
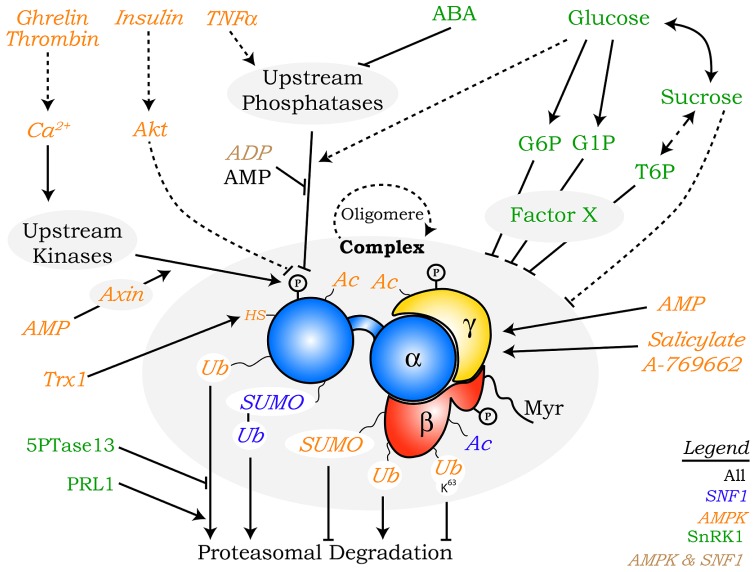
**Overview of the regulatory mechanisms controlling SNF1/AMPK/SnRK1 kinases described in this review.** Multiple factors regulate SNF1 (blue), AMPK (orange), and SnRK1 (green), some of which are conserved in all eukaryotes (black) or only in mammals and yeast (brown). In cases where a specific subunit is the known target of a particular regulatory mechanism this is indicated with a direct arrow to it, whereas in other cases regulation of the complex as a whole (“complex”) is indicated. Broken lines and full lines designate indirect links and direct connections, respectively. P, phosphorylation; Ac, acetylation; Ub, ubiquitination; Ub^K63^, ubiquitination through K63 chains; SUMO, small ubiquitin-like modifier; Myr, myristoylation; HS, reduced cysteine; Trx1, thioredoxin1.

Several aspects of the SnRK1 kinases are highly conserved, such as their core function as regulators of metabolism. Furthermore, plants also possess β and γ regulatory subunits that, together with the α catalytic subunits most probably form heterotrimeric complexes similar to the ones crystallized in other organisms (Amodeo et al., 2007; Polge and Thomas, 2007; Hardie, 2011; Xiao et al., 2011). On the other hand, the SnRK family has largely diverged and expanded, and plants have also evolved unique regulatory subunits, presumably to perform plant-specific functions (Halford et al., 2003; Polge and Thomas, 2007; **Figure 1**). In addition, although all Snf1/AMPKα/SnRK1α members require phosphorylation at a conserved activation loop threonine for their activity (**Figure 1**; Polge and Thomas, 2007; Hedbacker and Carlson, 2008; Hardie, 2011), the clear connection between such phosphorylation and differential kinase activity described for mammals and yeast is not well established in plants, suggesting additional regulatory mechanisms (Baena-Gonzalez et al., 2007; Fragoso et al., 2009; Nunes et al., 2013b; Rodrigues et al., 2013). Our aim is to provide a comprehensive review on the post-translational mechanisms that regulate SNF1/AMPK/SnRK1 kinases, some conserved across all eukaryotes and some specific for a particular member (**Figure 2**). These mechanisms most likely play a role in the swift regulation of kinase activity in response to stress. Less direct modes of regulation such as transcriptional control or alternative splicing are probably more important for SNF1/AMPK/SnRK1 complex composition in different tissues and developmental stages, and are beyond the scope of this review. Likewise, the detailed function of these kinases as well as the downstream mechanisms by which they regulate gene expression and protein function will not be covered here, as they have been extensively reviewed elsewhere (Polge and Thomas, 2007; Hedbacker and Carlson, 2008; McGee and Hargreaves, 2008; Hardie, 2011; Carling et al., 2012).

## STRUCTURE OF THE SNF1/AMPK/SnRK1 COMPLEXES

The SNF1/AMPK/SnRK1 protein kinases are conserved throughout all eukaryotes and share an αβγ heterotrimeric structure (**Figure 1**; Polge and Thomas, 2007; Hedbacker and Carlson, 2008; Carling et al., 2012). The catalytic α-subunit is composed of two parts, the kinase domain and the regulatory domain. The kinase domain displays a canonical fold with 11 sub-domains (Hanks and Hunter, 1995) and contains the activation loop (also called T-loop). The regulatory domain in yeast and mammals contains an auto-inhibitory sequence (AIS) which was shown to inhibit kinase activity (Pang et al., 2007; Chen et al., 2009). In plants, this region appears not to be inhibitory (Shen et al., 2009) and harbors an ubiquitin-associated (UBA) domain that was proposed to mediate the interaction with ubiquitinated proteins (Farras et al., 2001). In addition, these kinases possess a kinase-associated 1 (KA1) domain responsible for the interaction with the regulatory subunits and the upstream phosphatases (**Figure 1A**; Kleinow et al., 2000; Amodeo et al., 2007; Xiao et al., 2011; Rodrigues et al., 2013).

The function, in yeast and mammals, of the γ regulatory subunit is to control the activity of the α-catalytic subunit (Hedbacker and Carlson, 2008; Carling et al., 2012). This occurs through binding of adenylates to the cystathionine-β-synthase (CBS) domains (Bateman, 1997; **Figures 1A,B**).

The β-subunit acts as a scaffold keeping the α and the γ subunits together. This seems to be its primordial function, as the plant-specific β3 subunit, which contains only the scaffold part (the association with SNF1 complex, ASC domain; **Figure 1**), is able to complement the *gal83*Δ*sip1*Δ*sip2*Δ yeast mutant, devoid of all three β-subunits (Gissot et al., 2004). Nevertheless, these subunits contain also a carbohydrate binding motif (CBM, previously called glycogen binding domain, GBD) that, in AMPK and SNF1 Gal83 and Sip2, binds glycogen *in vitro* (Wiatrowski et al., 2004; McBride et al., 2009). The β-subunits also contain an N-terminal extension that is believed to facilitate association with downstream targets and determines trimer localization in yeast (Hedbacker and Carlson, 2008).

Even though SNF1/AMPK/SnRK1 subunits are globally conserved throughout eukaryotes, two atypical subunits exist in plants: the β3 subunit mentioned above and the βγ subunit (**Figure 1A**), a true γ-type subunit with an N-terminal extension containing a CBM (Lumbreras et al., 2001). In addition to SnRK1βγ, plants have a large family of γ-like subunits, including SnRK1γ (Ramon et al., 2013). However, in leaf mesophyll cells transiently overexpressing various subunit combinations, only SnRK1βγ interacts with β-subunits and assembles into a heterotrimeric complex. This observation, coupled to the fact that only SnRK1βγ complements *snf4*Δ (the SNF1 γ-subunit), suggests that SnRK1γ might not play a “canonical” γ-function (Ramon et al., 2013).

As illustrated in **Figure 1**, several heterotrimer compositions are possible *in vivo* in all eukaryotes (up to 12 in *Arabidopsis*). This is probably the first layer of regulation of these kinases, as for instance, different β-subunits within the SNF1 complex trigger differential localization (Hedbacker and Carlson, 2008).

## REGULATION BY PHOSPHORYLATION

Phosphorylation of a conserved threonine in the T-loop of the catalytic subunit (SnRK1α1^T175^/SnRK1α2^T176^; AMPKα2^T172^; Snf1^T210^; **Figure 1A**) is essential for SNF1/AMPK/SnRK1 activity (Stein et al., 2000; McCartney and Schmidt, 2001; Baena-Gonzalez et al., 2007; Shen et al., 2009; Crozet et al., 2010). Many protein kinases share this mode of regulation, which is presumably required for the proper alignment of ATP in order to allow its interaction with the catalytic lysine (Johnson, 2009). When grown in high glucose concentrations Snf1^T210^ is predominantly in the dephosphorylated state and the SNF1 complex is inactive. Upon shifting the yeast cells to a medium depleted of glucose, Snf1^T210^ becomes strongly phosphorylated, rendering the complex active (McCartney and Schmidt, 2001). Similarly to SNF1, the AMPK complex in mammals is activated by metabolic stresses that compromise ATP production or increase ATP consumption (Hardie, 2011), and displays appreciable activity only when phosphorylated on the conserved activation loop threonine residue (Stein et al., 2000). In plants, analyses of total cell extracts reveal no differences in the phosphorylation levels of the activation loop between control and stress conditions (Baena-Gonzalez et al., 2007; Fragoso et al., 2009; Coello et al., 2012; Rodrigues et al., 2013). This may suggest the involvement of additional phosphoresidues or other mechanisms for controlling activity upon stress. However, recent analyses of SnRK1 phosphorylation following size fractionation revealed higher T-loop phosphorylation when the catalytic subunit was incorporated into a complex (Nunes et al., 2013b). It is hence possible that stress promotes only the phosphorylation of the catalytic subunits that are assembled into a complex, and that analyses of total cellular SnRK1 have missed this fine level of regulation.

### UPSTREAM KINASES

The first report on the existence of an SNF1/AMPK/SnRK1 upstream kinase was in mammals in 1987 (Carling et al., 1987). However, the three yeast SNF1 activating kinases, Elm1, Tos3, and Sak1, were the first of the family to be identified by combining co-immunoprecipitation (Tos3 and Sak1 with Snf4), phylogenetic analyses (Elm1 clusters with Tos3 and Sak1), and genetics (triple *sak1*Δ*elm1*Δ*tos3*Δ shared the same phenotype than s*nf1*Δ; Hong et al., 2003). To some extent SNF1 activating kinases are functionally redundant, since all three need to be knocked out to prevent growth on alternative carbon sources. Nevertheless, they present stress-dependent preferences toward specific β-subunits of the SNF1 complex with a differential impact on the phosphorylation of downstream targets such as the Mig1 transcription factor (McCartney et al., 2005).

Sak1 is the primary SNF1 upstream kinase as it is the only one that associates strongly with Snf1 and *sak1*Δ is more affected in SNF1 activity than the *elm1*Δ and *tos3*Δ mutants (Hedbacker et al., 2004a; Elbing et al., 2006a). Sak1 is the only upstream kinase controlling the SNF1 complexes containing Gal83 as a β-subunit under low glucose (Hedbacker et al., 2004a; McCartney et al., 2005). In these conditions Gal83-SNF1 relocalizes from the cytoplasm to the nucleus, whilst Sip1-SNF1 relocalizes to the vacuolar membrane and Sip2-SNF1 remains cytoplasmic (Hedbacker and Carlson, 2008). Lack of Tos3 leads to a 20% decrease in SNF1 phosphorylation and activity during glucose starvation (Hong et al., 2003) having a noticeable effect on growth only when cells are continuously maintained on non-fermentable carbon sources (Kim et al., 2005). Finally, Elm1 was already known to phosphorylate and regulate a number of proteins necessary for proper cell morphogenesis and cell-cycle progression (Sreenivasan and Kellogg, 1999) before being identified as a SNF1 kinase (Hong et al., 2003).

The tumor suppressor liver kinase B1 (LKB1) was the first AMPK upstream kinase to be identified in mammals based on its sequence similarity with the yeast SNF1 activating kinases (Hawley et al., 2003; Woods et al., 2003a). LKB1 associates in a constitutively active complex (Sakamoto et al., 2004) with the pseudokinase STRAD and the adaptor protein MO25/CAB39 (Hawley et al., 2003). LKB1 is expressed in virtually all human tissues (Alessi et al., 2006), and it phosphorylates AMPK in response to an elevated AMP/ATP ratio (Woods et al., 2003a; Gowans et al., 2013). LKB1 has been implicated in numerous cellular processes including metabolism, cell cycle progression, cell polarity and embryogenesis, mostly due to its phosphorylation of AMPK and AMPK-related kinases (Alessi et al., 2006).

In certain cell types, like neurons, AMPKα^T172^ can also be phosphorylated by the Ca^2+^/calmodulin-dependent protein kinase kinase CaMKKβ, allowing the input of Ca^2+^-signals into the AMPK pathway (Hawley et al., 2005; Woods et al., 2005), independently of the adenine nucleotide ratios (Fogarty et al., 2010). Consistent with the lack of regulation by adenylates, the CaMKKβ–AMPK complexes are devoid of AMPKγ and contain only AMPKα and β-subunits (Anderson et al., 2008).

Finally, the transforming growth factor-β-activated kinase (TAK1) was shown to complement the yeast *elm1*Δ*sak1*Δ*tos3*Δ triple mutant suggesting that it might also be an AMPK upstream kinase (Momcilovic et al., 2006). Several lines of evidence support the role of TAK1 as an AMPK upstream kinase, in particular under conditions where reactive oxygen species and redox imbalance are generated (Xie et al., 2006; Chen et al., 2013).

The similarity between LKB1 and the *Arabidopsis* proteins GRIK2/1 (geminivirus Rep-interacting kinase 2 and 1), prompted the hypothesis that they may be upstream kinases of SnRK1 (Harthill et al., 2006). Their identification as SnAK1/2 (SnRK1 activating kinase 1 and 2) in *Arabidopsis* occurred shortly after, through functional complementation of the yeast *tos3Δpak1Δelm1Δ* triple mutant (Shen and Hanley-Bowdoin, 2006; Hey et al., 2007). This was substantiated by the demonstration that SnAKs can autophosphorylate and subsequently phosphorylate and activate recombinant SnRK1α *in vitro* (Shen et al., 2009; Crozet et al., 2010). Similarly to LKB1, SnAKs do not require Ca^2+^ and are insensitive to the CaMKK-specific inhibitor STO-609 (Shen et al., 2009). They also appear to be constitutively active and insensitive to AMP when assayed *in vitro* on the recombinant kinase domain of the SnRK1α subunit (Shen et al., 2009). This is consistent with the indirect effect of AMP on LKB1 activity mediated by the AMPKγ subunit and AXIN (see regulation by adenylates; Alessi et al., 2006; Gowans et al., 2013; Zhang et al., 2013). On the other hand, SnAKs autophosphorylate *in vitro* (Kong and Hanley-Bowdoin, 2002) on AtSnAK1^T153^/AtSnAK2^T154^ (Crozet et al., 2010), and this phosphorylation is required for their activity, as the corresponding phospho-mutant (S153A) and phospho-mimetic (S153D) variants are constitutively inactive and active, respectively (Crozet et al., 2010). In addition, they are phosphorylated and inhibited by SnRK1 on their T-loop (AtSnAK1^S260^/AtSnAK2^S261^) as part of a negative feedback loop to tightly control SnRK1 activity (Crozet et al., 2010). Interestingly, SNF1 phosphorylates Sak1 *in vitro*, and Tos3 phosphorylation by another kinase was inhibited by SNF1 (Elbing et al., 2006a), suggesting that this kinase cross-regulation might be conserved.

The relevance of SnAKs as SnRK1 upstream kinases remains to be assessed *in vivo*, as the only evidence for their function *in planta* is an overlap between SnAK expression and SnRK1 phosphorylation in the shoot apical meristem (Shen et al., 2009). SnAKs/GRIKs are only detected in actively proliferating tissue and in geminivirus-infected mature leaves (Shen et al., 2009), whilst SnRK1 phosphorylation is readily detected in other tissues such as mature non-infected leaves (Shen et al., 2009). It is possible that SnAKs/GRIKs phosphorylate SnRK1 also in mature leaves, where they simply accumulate to undetectably low levels, as suggested by their reported proteasomal degradation (Shen and Hanley-Bowdoin, 2006). However, this may also indicate the existence of additional upstream kinases. A study on rice reported the interaction of SnRK1 with CIPK15 (calcineurin B-like-interacting protein kinase 15), and several lines of evidence were presented to support a role of CIPK15 as a SnRK1 upstream kinase (Lee et al., 2009). Nevertheless, biochemical evidence demonstrating direct SnRK1 phosphorylation and activation by CIPK15 is still required to substantiate this conclusion. As the mammalian CaMKK can phosphorylate purified spinach SnRK1 *in vitro* (Sugden et al., 1999a), the possibility that endogenous Ca^2+^-dependent kinases like CIPKs or calcium-dependent protein kinases can serve as SnAKs is still open.

In addition to the phosphorylation of the conserved T-loop threonine, other phosphorylation events have been described in the AMPK α- and β-subunits (**Figure 2**; Mitchelhill et al., 1997; Woods et al., 2003b; Oppermann et al., 2009; Steinberg and Kemp, 2009), although in most cases their functional relevance is still unclear. On AMPKα, phosphorylation of S485 represses T172 phosphorylation and AMPK activity in response to activation of the Akt/PKB kinase by insulin and in response to PKA-mediated cAMP signaling (Horman et al., 2006; Hurley et al., 2006). PKA inhibits AMPKα by phosphorylating a second residue (S173) that also interferes with T172 phosphorylation (Djouder et al., 2009). S485 does not seem to be conserved in SnRK1α. On the other hand, whilst the AGC protein kinase family, to which PKA and Akt/PKB belong, is also present in plants (Garcia et al., 2012), there is no proof thus far for the existence of Akt/PKB (Dobrenel et al., 2011) or a cAMP activated kinase such as PKA (Gehring, 2010) in plants.

All three AMPK subunits were reported to be phosphorylated by the autophagy kinase Atg1/ULK1, leading to AMPK inactivation, although the effect of individual phosphorylation events was not further explored (Löffler et al., 2011). Given that AMPK controls autophagy by activating Atg1/ULK1 (Kim et al., 2011) this was proposed to establish a negative feedback loop to reset AMPK after activation of autophagy (Löffler et al., 2011).

Interestingly, Adi3 (AvrPto-dependent Pto-interacting protein3), a known suppressor of cell death triggered by pathogens in tomato, was shown to interact with SnRK1α1 and to phosphorylate the Gal83 β-subunit, thereby inhibiting the activity of the SnRK1 complex (Avila et al., 2012). It would be interesting to test whether such a phosphorylation is specific to pathogen attack or whether it occurs in response to other environmental, metabolic, or hormonal cues.

### UPSTREAM PHOSPHATASES

An increasing body of evidence indicates that the dephosphorylation step is crucial for regulating SNF1/AMPK/SnRK1 activity (Suter et al., 2006; Sanders et al., 2007; Rubenstein et al., 2008; Chandrashekarappa et al., 2011; Mayer et al., 2011; Oakhill et al., 2011; Xiao et al., 2011; Rodrigues et al., 2013).

To date, the best characterized protein phosphatases (PPs) of the SNF1/AMPK/SnRK1 family are those of yeast. The dephosphorylation of Snf1 catalytic subunit in response to the glucose signal is partly mediated by the PP1 phosphatase Glc7 that acts in a complex with the Reg1 regulatory subunit (Hedbacker and Carlson, 2008). Yeast cells lacking the Reg1 gene show a constitutively phosphorylated and active SNF1, even when glucose is available in the medium (McCartney and Schmidt, 2001). On the other hand the *glc7Δ* mutation is lethal (Cannon et al., 1994), partly because of excessive SNF1 activity. In addition to Glc7, recent studies demonstrate that Snf1 is also dephosphorylated by the type 2C phosphatase Ptc1 and the type 2A phosphatase Sit4 (Ruiz et al., 2011, 2013).

In low glucose conditions, Glc7-Reg1 is active toward the Mig1 transcription factor, whilst it is largely inactive toward the activation loop of Snf1. This indicates that glucose does not change Glc7-Reg1 activity, but rather controls SNF1 dephosphorylation indirectly by changing the ability of the activation loop to serve as a substrate for the phosphatase (Rubenstein et al., 2008). This conclusion is in agreement with the fact that the activatory effect of ADP on SNF1 is due to a conformational change in the complex that renders it resistant to phosphatase action (**Figure 2**; see regulation by adenylates; Chandrashekarappa et al., 2011; Mayer et al., 2011). However, recent evidence also supports direct regulation at the level of the catalytic subunit (Chandrashekarappa et al., 2013) and the phosphatase (Castermans et al., 2012). Regulated activation loop phosphorylation/dephosphorylation was shown to occur also independently of the regulatory subunits and trimer formation (Elbing et al., 2006b; Ruiz et al., 2012). Consistent with this, mutagenesis of the yeast γ-subunit residues predicted to contact bound adenylates had no effect on SNF1 activity, suggesting that in contrast to mammals (Oakhill et al., 2011; Xiao et al., 2011), adenylate binding to the γ-subunit in yeast is not required for its ability to protect Snf1 from dephosphorylation (Chandrashekarappa et al., 2013). The authors propose an alternative model in which phosphatase resistance is provided by ADP binding to the kinase active site, while incorporation of the phosphorylated catalytic subunit into the heterotrimer core is required for kinase activity (Chandrashekarappa et al., 2013). Finally, a recent study revealed that glucose exerts a more direct effect on phosphatase action, as it activates PP1 and PP2A post-translationally (Castermans et al., 2012).

AMPK has been shown to be dephosphorylated *in vitro* by PP1, PP2A, and the metal-dependent protein phosphatase PP2C (Carling et al., 1989; Davies et al., 1995), although PP1 and PP2C dephosphorylate AMPK more efficiently than PP2A (Garcia-Haro et al., 2010). Both PP2C and PP1 phosphatases are able to dephosphorylate AMPK *in vivo*, suggesting that the type of regulation might depend on the tissue and conditions of cell stimulation (Steinberg and Kemp, 2009; Carling et al., 2012). In mouse pancreatic β-cells, knockdown of the PP1α and PP1β catalytic subunits or R6, a regulatory subunit of PP1, caused a reduction in AMPKα^T172^ dephosphorylation following a low to high glucose switch (Garcia-Haro et al., 2010). The R6 subunit was also reported to physically interact with the AMPKβ subunits in co-immunoprecipitation and yeast two-hybrid experiments. On the other hand, in human embryonic kidney cells, RNAi of *Ppm1E*, but not *Ppm1A* (both PP2C members) resulted in increased AMPKα^T172^ phosphorylation and in assays with lysates of cells stably depleted of Ppm1F Ppm1E, a threefold increase in AMPKα^T172^ phosphorylation was observed (Voss et al., 2011).

In the case of plants, two PP2C phosphatases, ABI1 and PP2CA were recently reported to interact and dephosphorylate SnRK1α1 (Rodrigues et al., 2013), in agreement with earlier findings that human PP2C can dephosphorylate and inactivate spinach SnRK1α *in vitro* (Sugden et al., 1999a). These PP2Cs are well established negative regulators of the ABA pathway through their interaction with SnRK2s, and their repressive action is blocked by the ABA receptors upon ABA binding (Cutler et al., 2010). Hence, the regulation of SnRK1 by these PP2Cs allows not only the inactivation of SnRK1 in response to sugars, but also its activation in response to ABA (Rodrigues et al., 2013).

Other PPs have also been reported to interact with SnRK1, although the functional relevance of those interactions is unknown. Namely, another PP2C, PP2C74, interacts with SnRK1α2 *in vitro* and in yeast two-hybrid (Tsugama et al., 2012). In addition, a dual-specificity protein tyrosine phosphatase, PTPKIS1, was reported to interact with SnRK1α2 *in vitro* and in yeast two-hybrid (Fordham-Skelton et al., 2002). This phosphatase was later on shown to harbor a CBM domain that allows binding to starch *in vitro* and *in vivo* (Kerk et al., 2006) and was identified as the component responsible for the starch overaccumulation of the *sex4* mutant (Niittyla et al., 2006). SEX4/PTPKIS1 is chloroplastic and can bind and dephosphorylate phosphoglucans, suggesting that it regulates the initial steps of starch degradation at the granule surface (Niittyla et al., 2006; Kotting et al., 2009).

## REGULATION BY OTHER POST-TRANSLATIONAL MODIFICATIONS

Although T-loop phosphorylation is generally considered the main mechanism for regulating SNF1/AMPK/SnRK1 activity, several other post-translational modifications have been described, including acetylation, ubiquitination, SUMOylation, and myristoylation and oxidation (**Figure 2**).

### ACETYLATION

Sip2, a β-regulatory subunit of SNF1, was identified as a non-chromatin substrate of the nucleosome acetyltransferase of H4 complex (NuA4) in a yeast proteome microarray, and its acetylation was validated by *in vitro* activity assays and confirmed *in vivo* (Lin et al., 2009). Sip2 acetylation stabilizes its interaction with the Snf1 catalytic subunit thereby inhibiting it. On the other hand, Sip2 acetylation decreases gradually with cell age and Sip2 acetylation mimetics live longer, altogether suggesting that Sip2 acetylation extends lifespan through inhibition of SNF1 activity (Lu et al., 2011).

The AMPKα1 catalytic subunit was shown to be acetylated *in vitro* by the p300 acetyltransferase, but the *in vivo* confirmation of this modification awaits further studies (Lin et al., 2012, 2013). Additionally, a mass spectrometry analysis of AMPK subunits revealed that AMPKγ1 is acetylated on the N-terminus, with no other post-translational modifications detected in this subunit (Mitchelhill et al., 1997).

Even though none of the SnRK1 subunits have been reported to be acetylated, studies in mammals and yeast suggest that all three subunits could be subjected to this modification.

### UBIQUITINATION

In yeast, ubiquitination negatively modulates Snf1 stability, phosphorylation, and catalytic activity during growth on alternative carbon sources. Ubp8, a subunit of the histone modifier SAGA complex, was shown to deubiquitinate Snf1 (Wilson et al., 2011). Accordingly, when grown on galactose medium, the *ubp8Δ* strain showed lower Snf1 levels and Snf1^T210^ T-loop phosphorylation than the wild-type (WT), due to enhanced proteasome-mediated protein degradation (Wilson et al., 2011). On the other hand, Snf1 is SUMOylated (see below) and this modification promotes its proteasome-dependent degradation through the Slx5–Slx8 SUMO-targeted ubiquitin ligase (Simpson-Lavy and Johnston, 2013).

Al-Hakim et al. (2008) were the first to report the *in vivo* polyubiquitination of AMPKα1 and other AMPK-related kinases through unusual K^29^/K^33^-linked polyubiquitin chains. Although for the AMPK-related kinases ubiquitination was shown to interfere with T-loop phosphorylation and kinase activity (Al-Hakim et al., 2008), it remains to be determined whether AMPK activity is also similarly affected. Another study described the modulation of AMPK stability and activity by Cidea (cell death-inducing DFF45-like effector A)-mediated ubiquitination in brown adipose tissue (Qi et al., 2008). Cidea and AMPK form a complex *in vivo*, through a specific interaction with AMPKβ. *Cidea*-null mice accumulate higher levels of AMPK α-, β-, and γ-subunits with a consequent increment of AMPKα^T172^ phosphorylation and catalytic activity. Conversely, expression of Cidea promoted proteasomal degradation of the AMPK complex (Qi et al., 2008). On the other hand, mice deficient in the (UCH)-L3 deubiquitinating enzyme displayed increased AMPKα^T172^ phosphorylation and fatty acid oxidation, suggesting that ubiquitination might render AMPK more active (Setsuie et al., 2010). However, the effect might be indirect, since normal AMPK activity could not be restored by the short time replenishment of (UCH)-L3 expression within 4–6 days. Ubiquitination of AMPKβ with K^63^-linked chains, on the other hand, was shown to promote AMPKβ stability possibly through its allocation into inclusion bodies and subsequent protection from proteolytic turnover (Moreno et al., 2010). Nevertheless, this modification did not induce detectable changes in AMPK activity.

Interestingly, in plants, inactive kinase SnRK1α1^K48M^ and T-loop phosphorylation SnRK1α1^T175A^ mutant proteins accumulate to much higher levels than the WT SnRK1α1 protein (Baena-Gonzalez et al., 2007), suggesting that activity and phosphorylation are connected with the stability of the protein. In agreement with this view, SnRK1α1 is targeted for proteasomal degradation under low nutrient conditions in a myoinositol polyphosphate 5-phosphatase (5PTase13)-dependent manner (Ananieva et al., 2008). SnRK1α1 degradation appears to be mediated also by the DDB1-CUL4-ROC1-PRL1 E3 ubiquitin ligase, in which PRL1 is the putative substrate receptor of the complex (Lee et al., 2008). SnRK1α1 interacts with PRL1 (Bhalerao et al., 1999; Farras et al., 2001) and its degradation *via* the 26S proteasome is slower in *prl1* and *cul4cs* extracts than in the WT, accumulating to a higher extent in these mutants (Lee et al., 2008). In accordance, *prl1* exhibits a higher activation of SnRK1 in comparison to the WT (Bhalerao et al., 1999), and the activity of 3-hydroxy-3-methyl-glutaryl-CoA reductase, an enzyme inhibited by SnRK1 phosphorylation (Sugden et al., 1999b), is reduced in *prl1* seedlings (Flores-Perez et al., 2010). On the other hand, PRL1 was reported to compete with SKP1/ASK1 for binding SnRK1α1 and SnRK1α2. SKP1/ASK1 is a component of the SCF E3 ubiquitin ligase and although SnRK1 participates in the assembly of a proteasomal complex with this E3 ligase, there is also the possibility that SnRK1 degradation is mediated either by the SCF complex or the CUL4-DDB1 machinery upon varied conditions (Farras et al., 2001; Lee et al., 2008).

### SUMOylation

In yeast, a novel regulatory layer in SNF1 was uncovered through SUMOylation of its Snf1 catalytic subunit. In the presence of glucose, Snf1 is a target of the E3 SUMO ligase Mms21 which catalyzes the covalent attachment of SUMO at Snf1^K549^ in its C-terminal regulatory domain. SUMOylation can be reverted by the SUMO protease Ulp1 (Simpson-Lavy and Johnston, 2013). In the presence of glucose, SUMOylation inhibits Snf1 independently of T-loop phosphorylation, counteracting the glucose deprivation response. The authors suggest that upon SUMOylation an intramolecular conformational switch occurs due to the interaction between SUMOylated K549 with a SUMO-interacting motif located near the active site, promptly leading to an inactive conformation of Snf1. On the other hand, the SUMO tag targets Snf1 to ubiquitination *via* the SUMO-targeted E3 Ubiquitin ligase Slx5–Slx8, leading to degradation and attenuation of Snf1 levels in the cell as a response to glucose sensing.

The AMPK complex was also recently shown to be post-translationally regulated by SUMOylation (Rubio et al., 2013), underpinning previous results where AMPK subunits interacted with E2 SUMO conjugating enzyme in a yeast two-hybrid screen (Moreno et al., 2009). E3 SUMO ligase PIASy specifically modifies AMPKβ2 subunit with the SUMO2 isoform, leading to the formation of poly-SUMO2 chains. The authors used a hypersumoylable mutant (AMPKβ2^K262R^) to show that SUMOylation of the AMPKβ2 subunit enhances the activity of the AMPK heterotrimeric complex (α2β2γ1), based on increased T-loop phosphorylation of AMPKα2 and on increased phosphorylation of the target acetyl-CoA carboxylase. SUMOylation of AMPKβ2 competes with ubiquitination of the same subunit and antagonizes the ubiquitin-mediated degradation and hence reduction in activity of overall AMPK complex (Rubio et al., 2013).

In plants, SnRK1α1 was found to interact with the E2 SUMO conjugating enzyme and with the SUMO protease ESD4 in a yeast two-hybrid screen (Elrouby and Coupland, 2010). Furthermore, in the same study, SnRK1α1 was found to be SUMOylated with both SUMO1 and SUMO3 isoforms in a high-throughput assay in *Escherichia coli*. The fact that SnRK1α1 has high probability SUMO attachment sites (Elrouby and Coupland, 2010) and that SUMOylation has been implicated in the plant abiotic stress response (Castro et al., 2012) suggests that SUMOylation may be a conserved mechanism for controlling SNF1/AMPK/SnRK1 activity and stability.

### MYRISTOYLATION

In yeast, deficient protein *N*-myristoylation results in decreased resistance to nutrient deprivation, and the *N*-myristoylated protein Sip2 was found to be responsible for this phenotype (Ashrafi et al., 1998). Sip2 myristoylation was also implicated in the normal cellular life span (Lin et al., 2003). In young cells, myristoylated Sip2 is located at the plasma membrane and sequesters Snf4, the activating γ-subunit of the SNF1 complex. With aging, Sip2 re-localizes from the plasma membrane to the cytoplasm allowing Snf4–Snf1 entry into the nucleus. Nuclear SNF1 subsequently phosphorylates histone H3 and modifies chromatin structure (Lin et al., 2003).

An *N*-myristoylation consensus site is also present in the Sip1 β-subunit and it was shown to be required for Sip1 relocalization from the cytosol to the vacuolar membrane in response to various types of carbon stress. In glucose-grown cells, Sip1 localizes in the cytosol, and in response to carbon stress it re-localizes with Snf1 to the vacuolar membrane (Hedbacker et al., 2004b).

AMPKβ1 and β2 regulatory subunits were shown to be myristoylated *in vivo*, regulating AMPK activity and its subcellular localization (Mitchelhill et al., 1997; Oakhill et al., 2010). Myristoylation facilitates AMPK association with cellular membranes (Warden et al., 2001), and inhibition of myristoylation prevents the membrane association of AMPK in response to leptin (Suzuki et al., 2007) and glucose deprivation (Oakhill et al., 2010). A model has been proposed where the myristoyl group binds to a hydrophobic region within the complex, rendering AMPK inactive when ATP levels are high. The conformational change triggered by AMP binding exposes the myristoyl group thereby allowing the activation of the kinase and membrane association if required (Oakhill et al., 2010). This model can explain the increase in AMPK activity upon removal of the myristoylation site (Warden et al., 2001), but also the requirement of this modification for AMP-triggered stimulation of AMPKα^T172^ phosphorylation and AMPK maximal activation (Oakhill et al., 2010, 2011).

In *Arabidopsis, N*-myristoyltransferase (NMT) mutants exhibit various degrees of shoot apical meristem abnormalities, with lack of NMT1 causing growth arrest after germination (Pierre et al., 2007). The developmental arrest of *nmt1-1* mutants was caused by deficient myristoylation of a very small set of proteins, including the SnRK1β1 and β2 subunits. GFP fusions of either SnRK1β1 or SnRK1β2 localized to the plasma membrane, whereas G2A substitutions preventing myristoylation relocalized SnRK1β1 to the nucleus and SnRK1β2 to the cytoplasm. This provides a possible explanation for the fivefold increase in SnRK1 kinase activity measured in the *nmt1-1* mutant. Myristoylation of β1 or β2-subunits was proposed to sequester the complex to the plasma membrane acting as a negative regulator of the SnRK1 pathway, in accordance with the hypersensitivity to glucose of the *nmt1-1* mutant (Pierre et al., 2007).

### OXIDATION

A recent study revealed that AMPK activation is also regulated by the intracellular redox status (Shao et al., 2014). Oxidation of two key cysteine residues in the catalytic subunit interferes with the interaction between AMPK and LKB1, blocking AMPK phosphorylation and activity. Thioredoxin1, on the other hand, promotes AMPK activation by reducing these cysteine residues, acting as an essential cofactor during energy starvation.

## REGULATION BY ADENYLATES

To date, regulation by adenylates has been shown for all SNF1/AMPK/SnRK1 kinases (**Figure 2**; Sugden et al., 1999a; Mayer et al., 2011; Oakhill et al., 2011; Xiao et al., 2011), and excellent recent reviews are available for AMPK (Carling et al., 2012; Oakhill et al., 2012).

In yeast, AMP does not allosterically activate SNF1 (Wilson et al., 1996) but ADP protects it from dephosphorylation (Chandrashekarappa et al., 2011; Mayer et al., 2011). Although this protection was initially thought to be mediated through ADP binding to Snf4, more recent work indicates that the regulatory subunits are not required for this protection (Chandrashekarappa et al., 2013). The current hypothesis is that, after phosphorylating a substrate, ADP would remain in the active site and protect the kinase from dephosphorylation.

Adenylate regulation of AMPK is known for almost as long as the AMPK itself, providing the basis for its name (Carling et al., 1989). Adenylates regulate AMPK at several levels. Firstly, AMPK is allosterically activated by AMP through binding to its γ-subunit (**Figure 1B**; Carling et al., 1987; Scott et al., 2004), and this activation has been confirmed to be specific to AMP (Gowans et al., 2013). Secondly, AMP binding to the γ-subunit of the AMPK complex increases its ability to serve as a substrate for an upstream kinase (Hawley et al., 1995; Oakhill et al., 2010), recently demonstrated to be LKB1 and not CaMKKβ (Gowans et al., 2013). A third protein, AXIN, associates with LKB1 and enhances its interaction with AMP-bound AMPK, explaining why only LKB1-dependent phosphorylation of AMPK is stimulated by AMP (Zhang et al., 2013). Thirdly, binding of the low energy adenylates ADP and AMP to the γ-subunit confers a conformation to the AMPK complex that makes it recalcitrant to dephosphorylation and inactivation by phosphatases (Davies et al., 1995; Suter et al., 2006; Oakhill et al., 2011; Xiao et al., 2011). ADP binding was proposed to be the major factor protecting AMPK from dephosphorylation (Xiao et al., 2011). More recent work questioned this hypothesis by showing that AMP, in its physiological concentration range, has a stronger effect than ADP on AMPK protection against phosphatases (Gowans et al., 2013).

The AMPKγ subunit harbors four CBS domains (**Figures 1A,B**; Bateman, 1997), two of which binding adenylates reversibly (1 and 3; Scott et al., 2004; Xiao et al., 2007) and one (4) seemingly binding AMP non-exchangeably (Xiao et al., 2011). Although initially proposed to have different functions in allosteric activation and protection from phosphatases (Xiao et al., 2011), two studies suggest that the three adenylate binding sites (1, 3, and 4) are equally important for both functions (Oakhill et al., 2010; Gowans et al., 2013). It is also important to note that without AMPKβ myristoylation, none of these events can occur (Oakhill et al., 2010).

In the case of plants, the adenylate sensitivity was assessed only on a purified SnRK1 complex from spinach leaves. It was observed that AMP protects from dephosphorylation when the purified complex is incubated with recombinant mammalian PP2C (Sugden et al., 1999a). Nothing is known about the mechanism underlying this effect but as all the subunits of the trimeric complex are conserved in eukaryotes and the plant subunit complements the corresponding yeast mutant (Polge and Thomas, 2007), it is reasonable to assume that the mechanism might be similar. On the other hand, the kinase domain is even more conserved, so a direct protective effect of ADP upon remaining on the active site is also possible.

## REGULATION BY SUGARS AND OTHER METABOLITES

In yeast, SNF1 is activated in response to low glucose concentrations and other stresses and quickly inactivated by the addition of glucose (reviewed in Hedbacker and Carlson, 2008). Interestingly, the glucose analog 2-deoxyglucose, which is phosphorylated but not further metabolized, also inhibits SNF1, whereas 6-deoxyglucose, which cannot be phosphorylated, has no effect (Hedbacker and Carlson, 2006). These observations may be explained by a rapid depletion of cellular ATP due to the phosphorylation of 2-deoxyglucose (Wilson et al., 1996). They may also suggest that glucose phosphorylation is needed for SNF1 repression, in agreement with the interaction of hexokinase2 with the Glc7–Reg1 complex for SNF1 repression (Moreno et al., 2005). Hexokinase2 activity in turn is repressed by trehalose-6-phosphate (T6P) and T6P synthase 1 (TPS1) to control the influx of glucose into glycolysis and prevent an overconsumption of ATP and metabolic arrest (Thevelein and Hohmann, 1995).

In mammals, high glucose concentrations inhibit AMPK activity (Itani et al., 2003; Minokoshi et al., 2004). However, high glucose concentrations do not always result in detectable changes in the adenylate charge, suggesting other modes of repression of AMPK activity independent of adenylates (Itani et al., 2003). Supporting this, glucose has been reported to have a direct activatory effect on PP1 and PP2A (Ravnskjaer et al., 2006; Castermans et al., 2012). Amino acids like leucine or glutamine also negatively regulate AMPK (Gleason et al., 2007). Citrate, produced by the TCA cycle in mitochondria, has been shown to inhibit AMPK activity in the rat hypothalamus during fasting (Cesquini et al., 2008). Furthermore, PP2A was reported to mediate palmitate-induced AMPK inhibition in mice fed with a high fat diet (Wu et al., 2007). Glycogen, particularly preparations with a high degree of branching, binds to β-subunits of AMPK leading to an allosteric inhibition of kinase activity. This may suggest that AMPK may sense not only the energy immediately available but also the availability of energy reserves (McBride et al., 2009).

In plants, T6P has arisen as one of the major regulators of SnRK1. T6P is found in trace amounts in most plants, where it is considered to function as a signaling molecule (Schluepmann et al., 2012). T6P accumulation is highly correlated with sucrose levels (**Figure 2**) and hence T6P has been proposed to relay information about carbohydrate availability to other signaling pathways (Lunn et al., 2006). Inhibition of SnRK1 activity from *Arabidopsis* seedling extracts by T6P was observed at concentrations in the micromolar range. This inhibition was also observed in extracts from different *Arabidopsis* tissues and other plants (spinach, broccoli, and cauliflower), with the exception of fully mature leaves (Zhang et al., 2009), indicating that SnRK1 regulation depends on the developmental stage and probably the type of tissue (Zhang et al., 2009; Martinez-Barajas et al., 2011). Interestingly, no effect of T6P was observed in the activities purified from yeast, nematodes, flies, or human liver, suggesting that this effect is plant specific. Immunoprecipitated or anion-exchange chromatography purified SnRK1 is catalytically active but is no longer inhibited by T6P. However, the inhibition can be restored by supplementing the supernatant from immunoprecipitated seedling extracts indicating that an intermediary factor separable from SnRK1 activity is necessary for inhibition of SnRK1. The need for this intermediary factor is also suggested by the fact that mature leaf supernatant could not restore T6P inhibition (Zhang et al., 2009). Inhibition of SnRK1 by T6P has also been observed in potato tubers fed with trehalose (Debast et al., 2011), in wheat grain (Martinez-Barajas et al., 2011) and in sugar cane (Wu and Birch, 2010). In agreement with this, plants accumulating elevated T6P levels through overexpression of the *E. coli* T6P synthase *otsA* presented an opposite transcriptional profile to that triggered by SnRK1 activation (Zhang et al., 2009). Similar results were observed in transgenic potato tubers (Debast et al., 2011). During wheat grain development, SnRK1-induced and SnRK1-repressed marker gene expression changes in the different tissues of the seed are correlated with changes in T6P levels, further supporting SnRK1 inhibition by T6P (Martinez-Barajas et al., 2011). A similar correlation was observed in *Arabidopsis* seedling extracts (Nunes et al., 2013a). It is also noteworthy that seedling growth arrest on high concentrations of trehalose, due to T6P accumulation, is rescued by overexpression of SnRK1 (Delatte et al., 2011). This suggests that T6P prevents growth on trehalose through the inhibition of SnRK1. Consistent with the inhibition of SnRK1 by T6P, plants silenced for SnRK1α1 and SnRK1α2 senesce early (Baena-Gonzalez et al., 2007), opposite to plants overexpressing SnRK1α1 or to plants with low T6P levels which have delayed senescence (Baena-Gonzalez et al., 2007; Wingler et al., 2012).

As explained above, T6P is a major regulator of glycolysis in yeast through repression of hexokinase 2 (Thevelein and Hohmann, 1995). However, plant hexokinase activity seems not to be affected by up to 5 mM concentrations of T6P (Eastmond et al., 2002).

In addition to T6P, SnRK1 activity is inhibited by other sugars, such as glucose-6-phosphate (G6P), glucose-1-phosphate (G1P), glucose, and sucrose (**Figure 2**). SnRK1 is repressed by G6P in spinach (Toroser et al., 2000), sugar cane (Wu and Birch, 2010), and *Arabidopsis* (Nunes et al., 2013b). G1P inhibits SnRK1 more strongly than G6P and synergistically with T6P (Nunes et al., 2013b). Strikingly, a combination of immunoprecipitation and SnRK1 activity assays suggest that both G1P and G6P, likewise T6P, inhibit SnRK1 *via* an intermediary factor that is separable from SnRK1 (Nunes et al., 2013b). Supply of exogenous non-phosphorylated glucose and sucrose (5–50 mM) to seedlings and mature leaves also inhibits SnRK1 activity, as suggested by gene expression analyses (Baena-Gonzalez et al., 2007). On the other hand, several studies have reported an induction of SnRK1 activity by sucrose (Bhalerao et al., 1999; Jossier et al., 2009) or a SnRK1-dependent activation of gene expression or enzyme activity by sucrose (Purcell et al., 1998; Tiessen et al., 2003; Kolbe et al., 2005; Jossier et al., 2009). Such effect may be due to the heterotrophic nature of the material employed, in which SnRK1 may be regulated differently than in autotrophic leaves, or to the high sugar concentrations used, which may trigger stress and defense responses (Wingler and Roitsch, 2008). Finally, inhibition of SnRK1 by ribose-5-phosphate (Piattoni et al., 2011; Nunes et al., 2013b) and ribulose 5-phosphate (Nunes et al., 2013b) was also observed; however, this inhibition is probably indirect, due to a decrease of ATP availability through ATP-dependent ribulose-1,5-bisphosphate synthesis (Nunes et al., 2013b).

## REGULATION BY OLIGOMERIZATION

The crystal structure of the so called Bateman domain (a CBS-domain pair; **Figure 1A**) of yeast Snf4 (Rudolph et al., 2007) and of the γ-subunit of mammalian AMPK (Day et al., 2007) revealed the formation of dimers, which in yeast also formed *in vivo* (Rudolph et al., 2007). Crystallographic dimers were likewise obtained for the kinase domain of Snf1 (Rudolph et al., 2005; Nayak et al., 2006), and co-immunoprecipitation assays of differently tagged catalytic subunits in yeast cells confirmed the existence of these dimers *in vivo* (Nayak et al., 2006). The dimerization interface in SNF1 and AMPK is characterized by extensive hydrophobic interactions, involving both conserved and non-conserved residues around the activation loop (Nayak et al., 2006; Scholz et al., 2009). However, the exact interface of dimerization remains to be determined, as there are conflicting reports amongst the known structures (Amodeo et al., 2007; Townley and Shapiro, 2007; Xiao et al., 2007).

Importantly, oligomerization of whole heterotrimeric complexes has also been reported. Heterotrimers of truncated SNF1 from *Schizosaccharomyces pombe* and *Saccharomyces cerevisiae* expressed in bacteria formed crystallographic dimers (Amodeo et al., 2007; Townley and Shapiro, 2007), which for *Schizosaccharomyces pombe* were also found in solution. Even though the crystals of truncated AMPK complex did not show evidence of dimerization (Xiao et al., 2007), untagged, full-length and enzymatically competent AMPK heterotrimers purified from bacteria did form dimers (Riek et al., 2008). Dimers of AMPK heterotrimers and even higher order oligomers were also detected in cellular extracts by Blue Native PAGE (Scholz et al., 2009). Likewise, gel filtration chromatography data for AMPK revealed a higher molecular weight than expected (Riek et al., 2008). This can be merely due to a global non-spherical shape of the heterotrimer, but it has also been proposed to correspond to higher order complexes (Rudolph et al., 2007; Riek et al., 2008).

Dimerization appears to be a reversible concentration-dependent process that can occur both *in vitro* and *in vivo* in mammalian cells and it may be particularly important in specific subcellular *loci* where the kinases are highly concentrated (Riek et al., 2008; Scholz et al., 2009). However, how oligomerization impacts on kinase activity is not understood. The formation of higher order oligomers was associated to an inactive state of the AMPK complex (Scholz et al., 2009), which upon activation would disassemble into dimeric and monomeric units of the heterotrimeric complex. However, the activation loop of Snf1 becomes inaccessible for phosphorylation by upstream kinases when the catalytic subunits form dimers, suggesting that dimers of heterotrimers would also be inactive (Nayak et al., 2006).

When considering the total number of α, β, and γ subunits present in plants, at least 12 heterotrimers can be formed (**Figure 1A**), but this number increases if alternatively spliced variants are taken into account. The theoretical molecular weight of such heterotrimers ranges from 118 to 165 kDa (Nunes et al., 2013b), but immunoprecipitation of the SnRK1 complex *in vivo*, coupled to size fractionation, revealed the presence of both catalytic subunits in fractions of higher molecular weight (Nunes et al., 2013b). Supporting the formation of higher order complexes, the maize βγ-subunit was shown to homodimerize *in vitro* and *in vivo* (Lopez-Paz et al., 2009). However, it is not yet known whether this impacts on SnRK1 activity or if it is related to specific functions of the βγ-subunit.

## REGULATION BY DRUGS AND XENOBIOTICS

AMPK is known to be affected by an array of chemicals of synthetic (drugs) and natural (xenobiotics) origin, many of which are employed in the treatment of diabetes, obesity and cancer. We will briefly review the effects and mechanisms of action of the best characterized ones, as the regulation of AMPK by drugs and xenobiotics has been specifically reviewed elsewhere recently (Hardie et al., 2012a).

Most drugs or xenobiotics activate AMPK indirectly by blocking ATP production, either by inhibiting glycolysis (2-deoxyglucose) or oxidative phosphorylation. The latter is the case of mitochondrial poisons like oligomycin and dinitrophenol (Hawley et al., 2010), phenobarbital (Rencurel et al., 2005), drugs used in the treatment of type 2 diabetes like phenformin (Owen et al., 2000), rosiglitazone (Fryer et al., 2002), and several plant products considered to have health-promoting properties like berberine (Turner et al., 2008), resveratrol (Baur et al., 2006), or curcumin (Lim et al., 2009).

There are also chemicals that interact with upstream components of the AMPK pathway and indirectly promote its activation, like A23187 and other Ca^2+^ ionophores, that increase intracelular Ca^2+^ concentration, activating CaMKKβ (Hawley et al., 2005, 2010).

Other chemicals activate AMPK through a direct interaction. This is the case for metformin, which interacts with AMPKγ (Zhang et al., 2012) and compound A-769662 and salicylate, which activate AMPK by a similar mechanism involving the β1 and γ subunits. This results in allosteric activation of AMPK and its protection against T172 dephosphorylation (Scott et al., 2008; Hawley et al., 2012). Other small molecule AMPK activators are the related PT1 and C24 (Pang et al., 2008; Li et al., 2013). PT1 was found in a chemical library screen with inactive truncated human AMPK (Pang et al., 2008). It was proposed to bind near the autoinhibitory domain and directly relieve autoinhibition. Finally, a widely used AMPK activator is 5-aminoimidazole-4-carboxamide riboside (AICAR). AICAR is an adenosine analog which is a substrate for adenosine transporters and kinases. It is phosphorylated to the AMP analog, ZMP, and it is capable of activating AMPK (Corton et al., 1995), by binding to γ-subunit in a similar manner to AMP (Day et al., 2007). Finally, it is surprising that only one chemical, compound C, has been shown to inhibit AMPK by targeting the kinase domain (Zhou et al., 2001).

## REGULATION BY HORMONES

With the exception of the obligate intracellular parasite *Encephalitozoon cuniculi*, SNF1/AMPK/SnRK1 are ubiquitously present in eukaryotes, from simple unicellular organisms to complex multicellular ones (Hardie, 2011). These systems have therefore acquired the ability to be regulated by hormones and systemic signals, which in multicellular organisms is essential for the proper coordination of energy balance at the whole-organism level.

In mammals, AMPK activity is coordinated at the whole-body level through regulation by several hormones and cytokines, including leptin, adiponectin, resistin, ghrelin, insulin, glucagon-like peptide-1, glucocorticoids, inflammatory mediators, and thyroid hormone T3 and T4 (Hardie, 2010; Lim et al., 2010). Noteworthy, the effect of some hormones on AMPK activity depends on the tissue, and, for example, leptin activates AMPK in adipose tissue and the liver, whilst repressing it in the heart and the hypothalamus (Lim et al., 2010). Although in most cases the precise mechanisms underlying hormonal regulation of AMPK are unknown (Steinberg and Kemp, 2009; Lim et al., 2010), it is well established that insulin inhibits AMPK in cardiac tissue by activating the Akt/PKB kinase, which can phosphorylate AMPKα^S485^, thus leading to reduced phosphorylation at AMPKα^T172^ (Horman et al., 2006). In the case of thrombin, activation appears to occur through induction of Ca^2+^ signaling and CaMKKβ activation (Stahmann et al., 2006). Interestingly, chronic TNFα treatment in muscle cells suppresses the AMPK pathway through the induction of the repressor PP2C (Steinberg et al., 2006), suggesting that a connection between hormone signals and energy signaling through the repressive PP2Cs might be conserved in multicellular eukaryotes (see below).

In the case of plants, an increasing number of studies link SnRK1 to the ABA phytohormone (**Figure 2**). SnRK1 appears to play a central role in processes well known to be under ABA control, such as seed maturation and germination (Lu et al., 2007; Radchuk et al., 2010; Tsai and Gazzarrini, 2012). Furthermore, *Arabidopsis* plants overexpressing SnRK1α1 are hypersensitive to ABA during germination and early seedling development (Jossier et al., 2009; Tsai and Gazzarrini, 2012), consistent with the phosphorylation by SnRK1α1 of FUSCA3, a central transcription factor regulating seed maturation (Tsai and Gazzarrini, 2012). Recent work demonstrated that in mature photosynthetic tissues ABA activates SnRK1 through inhibition of its negative regulators, the 2C-type phosphatases ABI1 and PP2CA (Rodrigues et al., 2013). This may allow the complementation of the ABA response with a more general one triggered by SnRK1 and directed toward a metabolic and transcriptional reprograming. Additionally, the presence of ABA may potentiate SnRK1 signaling by blocking its inactivation and may allow SnRK1 activation in distant tissues not directly exposed to energy stress. Interestingly, ABA represses SnRK1 signaling *via* plant-specific SnRK1A-interacting negative regulators during germination and early seedling growth (Lin et al., 2014) and induces SnRK1 degradation in wheat roots (Coello et al., 2012). This suggests the effect of ABA may differ between autotrophic and heterotrophic tissues in a similar manner as animal hormones control AMPK in opposite manner in different tissues (Lim et al., 2010).

## CONCLUDING REMARKS

The regulation of the SNF1/AMPK/SnRK1 kinases is highly complex, involving, amongst others, multiple post-translational modifications of the catalytic and regulatory subunits, direct and indirect metabolic and hormonal control, and formation of higher order complexes (**Figure 2**). Regulation is exerted by universal signals like adenylates and sugars as well as by more specific signals like hormones or particular subunits that have evolved to regulate these kinases at the whole organism-level and possibly to serve organism-specific functions. Despite the conservation of some regulatory aspects, such as T-loop phosphorylation by homologous upstream protein kinases, the clear connection established for SNF1 and AMPK between T-loop phosphorylation, adenylate sensing, and kinase activity has not been fully established in plants where additional regulatory mechanisms may be operating.

On the other hand, the possible interconnection between the different modes of regulation is thus far unknown. Post-translational modifications might have an effect, e.g., on T-loop phosphorylation, either repressing it similarly to what has been described for ubiquitination of AMPK-related kinases or promoting it similarly to what has been described for SUMOylation of the AMPKβ subunit. Furthermore, the functional outcome of a particular mode of regulation might be rather complex and derived from several factors. An example of this is myristoylation of AMPKβ, which is necessary for the adenylate regulation of AMPK, but also for the membrane localization of the complex.

With regard to regulation by metabolites, hormones, and other compounds, mechanistic knowledge is lacking in most cases. Whether or not the effect of these compounds is direct, indirect involving changes in other metabolites or Ca^2+^ fluxes, or indirect involving unknown protein factors as reported for T6P in plants, remains to be determined. Moreover, the actual components transducing these signals to SNF1/AMPK/SnRK1 remain to be identified.

One major challenge lies in the heterogeneity of SNF1/AMPK/SnRK1 complexes. Subunit composition may change in response to specific conditions and may be unique to particular subcellular compartments, tissues or developmental stages. Subunit composition may determine the function of the complex as well as its mode of regulation. Therefore, strategies that allow monitoring specific complexes will ultimately be required for full characterization and understanding of these kinases. Different SNF1/AMPK/SnRK1 complexes are also likely to be recognized and controlled by different upstream regulators, as suggested by the rising number of kinases and phosphatases regulating AMPK and the specificity of some of these to particular tissues.

Full understanding of plant SnRK1 will also require the identification of further upstream regulatory components as well as a better characterization of their effects. In addition, the passive role of these upstream components, traditionally regarded as being constitutively active should be revisited, as an increasing body of evidence supports metabolic and hormonal regulation at least of the SNF1/AMPK/SnRK1 phosphatases. Identification and characterization of the upstream regulators may also be crucial for understanding the connection of these signaling cascades to other important pathways, as demonstrated for the dual role of ABI1/PP2CA phosphatases in SnRK1 and ABA signaling.

## Conflict of Interest Statement

The authors declare that the research was conducted in the absence of any commercial or financial relationships that could be construed as a potential conflict of interest.

## ABBREVIATIONS

5PTase: myoinositol polyphosphate 5-phosphatase
ABA: abscisic acid
ABI: ABA insensitive
Adi: AvrPto-dependent Pto-interacting protein
AICAR: 5-aminoimidazole-4-carboxamide riboside
AIS: auto-inhibitory sequence
AMPK: AMP-activated protein kinase
ASC: association with SNF1 complex
ASK1: *Arabidopsis* SKP1-like1
Atg: autophagy
AXIN: axis inhibitor
βIS: β-interacting sequence
CaMKK: Ca^2+^/calmodulin-dependent protein kinase kinase
cAMP: cyclic AMP
CBS: cystathionine-β-synthase
CBM: carbohydrate binding motif
CIPK: calcineurin B-like-interacting protein kinase
Cidea: cell death-inducing DFF45-like effector A
CUL4: cullin 4
DDB1: damaged DNA binding 1
Elm1: elongated morphology 1
ESD4: early in short day 4
G1P: glucose-1-phosphate
G6P: glucose-6-phosphate
Gal83: galactose metabolism 83
GBD: glycogen binding domain
Glc7: glycogen 7
GRIK: geminivirus Rep-interacting kinase
KA1: kinase-associated 1
LKB1: liver kinase B1
Mig1: multicopy inhibitor of GAL gene expression 1
Mms21: methyl methanesulfonate sensitivity 21
MO25: mouse protein 25
NMT: *N*-myristoyltransferase
NuA4: nucleosome acetyltransferase of H4 complex
PAGE: polyacrylamide gel electrophoresis
PIASy: protein inhibitor of activated STAT y
PK: protein kinase
PP: protein phosphatase
Ppm: protein phosphatase Mg^2+^/Mn^2+^ dependent
PRL1: pleiotropic regulatory locus 1
Ptc1: phosphatase type 2C
PTPKIS1: protein tyrosine phosphatase kinase interaction sequence 1
R6: regulatory subunit 6 (protein phosphatase 1)
Reg1: resistance to glucose repression 1
ROC1: regulator of cullins 1
SAGA: Spt–Ada–Gcn5 acetyltransferase
Sak1: Snf1 activating kinase 1
SCF: Skp1-cullin-F box
SEX4: starch excess4
Sip1/2: SNF1-interacting protein 1/2
Sit4: suppressor of initiation of transcription4
SKP1: S-phase kinase-associated protein 1
Slx5/8: synthetic lethal of unknown [X] function 5/8
Snf: sucrose non-fermenting
SnAK: SnRK1 activating kinase
SnRK: SNF1-related protein kinases
STO-609: 7-oxo-7H-benzimidazo[2,1-a]benz[de]isoquinoline-3-carboxylic acid – acetic acid
STRAD: STE20-related kinase adaptor
SUMO: small ubiquitin-like modifier
T-loop: activation loop
T6P: trehalose-6-phosphate
TAK1: transforming growth factor-β-activated kinase
TNFα: tumor necrosis factor α
Tos3: target of SBF
TPS: T6P synthase
UBA: ubiquitin-associated
Ubp8: ubiquitin-specific processing protease
(UCH)-L3: ubiquitin carboxyl-terminal esterase L3
ULK1: unc-51 like autophagy activating kinase 1
Ulp1: ubiquitin-like protein
WT: wild-type
ZMP: AICA monophosphate.
